# Baculovirus-Mediated miR-214 Knockdown Shifts Osteoporotic ASCs Differentiation and Improves Osteoporotic Bone Defects Repair

**DOI:** 10.1038/s41598-017-16547-3

**Published:** 2017-11-24

**Authors:** Kuei-Chang Li, Yu-Han Chang, Mu-Nung Hsu, Shih-Chun Lo, Wan-Hua Li, Yu-Chen Hu

**Affiliations:** 1 Department of Chemical Engineering, National Tsing Hua University, Hsinchu, 300 Taiwan; 2 Center for Tissue Engineering, Chang Gung Memorial Hospital, Taoyuan, 333 Taiwan; 3 Department of Orthopaedic, Chang Gung Memorial Hospital, Taoyuan, 333 Taiwan

## Abstract

Osteoporotic patients often suffer from bone fracture but its healing is compromised due to impaired osteogenesis potential of bone marrow-derived mesenchymal stem cells (BMSCs). Here we aimed to exploit adipose-derived stem cells from ovariectomized rats (OVX-ASCs) for bone healing. We unraveled that OVX-ASCs highly expressed miR-214 and identified 2 miR-214 targets: *CTNNB1* (β-catenin) and *TAB2*. We demonstrated that miR-214 targeting of these two genes blocked the Wnt pathway, led to preferable adipogenesis and hindered osteogenesis. As a result, OVX-ASCs implantation into OVX rats failed to heal critical-size metaphyseal bone defects. We further engineered the OVX-ASCs with a novel Cre/loxP-based hybrid baculovirus vector that conferred prolonged expression of miR-214 sponge. Gene delivery for miR-214 sponge expression successfully downregulated miR-214 levels, activated the Wnt pathway, upregulated osteogenic factors β-catenin/Runx2, downregulated adipogenic factors PPAR-γ and C/EBP-α, shifted the differentiation propensity towards osteogenic lineage, enhanced the osteogenesis of co-cultured OVX-BMSCs, elevated BMP7/osteoprotegerin secretion and hindered exosomal miR-214/osteopontin release. Consequently, implanting the miR-214 sponge-expressing OVX-ASCs tremendously improved bone healing in OVX rats. Co-expression of miR-214 sponge and BMP2 further synergized the OVX-ASCs-mediated bone regeneration in OVX rats. This study implicates the potential of suppressing miR-214 by baculovirus-mediated gene delivery in osteoporotic ASCs for regenerative medicine.

## Introduction

Bone is a dynamic tissue that is continuously remodeled through the action of bone-forming osteoblasts and bone-resorbing osteoclasts^1^. Osteoporosis results from dysregulated bone turnover, leading to increased vulnerability to fracture^2,3^. However, osteoporotic fracture healing is often delayed and compromised. Although drugs for osteoporosis management are available, each drug has its own limitations^4^. Meanwhile, less attention is drawn towards bone repair following fractures in osteoporotic patients.

Upon bone fracture, bone marrow-derived mesenchymal stem cells (BMSCs) are recruited to injury site and differentiate into osteoblasts to orchestrate the healing process. As such, tissue engineering approach exploiting BMSCs to heal osteoporotic fractures is appealing. However, in ageing osteoporotic patients, BMSCs tend to differentiate into adipocytes rather than osteoblasts, leading to progressive fat accumulation and bone loss^5,6^. Such biased BMSCs differentiation arises from attenuated Wnt signaling^7^. Wnt pathway inhibits adipogenic factors (e.g. C/EBP-α and PPAR-γ) while promotes crucial osteogenic factors (e.g. Runx2 and osterix (Osx))^8^, but antagonizing Wnt signaling cascade shifts BMSCs commitment to adipocyte^9^.

MicroRNA (miRNA) are small RNAs that regulate cellular events by binding to the 3′-untranslated region (UTR) of target mRNAs^10^ and a number of miRNAs have been correlated with BMSCs differentiation and osteoporosis. Recently, miR-214 was found to be highly expressed in bone specimen from aged osteoporotic patients with fractures^11^, suggesting the correlation of miR-214 overexpression with poor bone repair in osteoporotic patients. We also uncovered aberrant miR-214 overexpression in BMSCs isolated from ovariectomized (OVX) rats (OVX-BMSCs), and suppressing miR-214 with miR-214 sponge (RNA that contains complementary binding sites to miR-214) promotes OVX-BMSCs osteogenesis and augments the ability of OVX-BMSCs to heal critical-size bone defects in osteoporotic rats^12^. Although OVX-BMSCs can be engineered to heal osteoporotic bone defects, adipose-derived stem cells (ASCs) have become attractive for bone regeneration because ASCs can be isolated in large quantities by liposuction^13–15^. However, ASCs are inferior to BMSCs in osteogenic differentiation capability, often resulting in delayed or incomplete repair of large bone defects^16,17^. To overcome this problem, we have developed Cre/loxP-based hybrid baculovirus (BV) vector system comprising two BV: one expressing Cre recombinase and the other substrate BV harboring the transgene cassette flanked by loxP sites^18^. After co-transduction of ASCs with the two BV, the expressed Cre recognizes the loxP sequences, excises the transgene cassette off the substrate BV genome, thereby leading to the formation of episomal DNA minicircle encompassing the transgene within the cells. Such hybrid BV system enables sustained transgene expression^19^ and improves ASCs differentiation and bone healing *in vivo* ^12^.

Despite the promise of ASCs for bone healing, whether osteoporotic ASCs exhibit more favorable adipogenic differentiation vs. osteogenic differentiation and the underlying mechanism remain unknown. Also, whether osteoporotic ASCs can be used to heal osteoporotic bone defects has yet to be explored. Using ASCs isolated from OVX rats (OVX-ASCs), here we investigated the miR-214 expression level, adipogenesis/osteogenesis preference, molecular pathway and how miR-214 affects the differentiation of surrounding OVX-BMSCs *in vitro* and bone healing in OVX rats. We further exploited the Cre/loxP-based BV persistently expressing miR-214 sponge to engineer OVX-ASCs, so as to knockdown intracellular and exosomal miR-214 levels, reverse the differentiation preference, substantiate osteogenesis and ameliorate bone healing in OVX rats.

## Results

### miR-214 regulates the switching of adipogenesis and osteogenesis in OVX-ASCs

**Figure 1:**
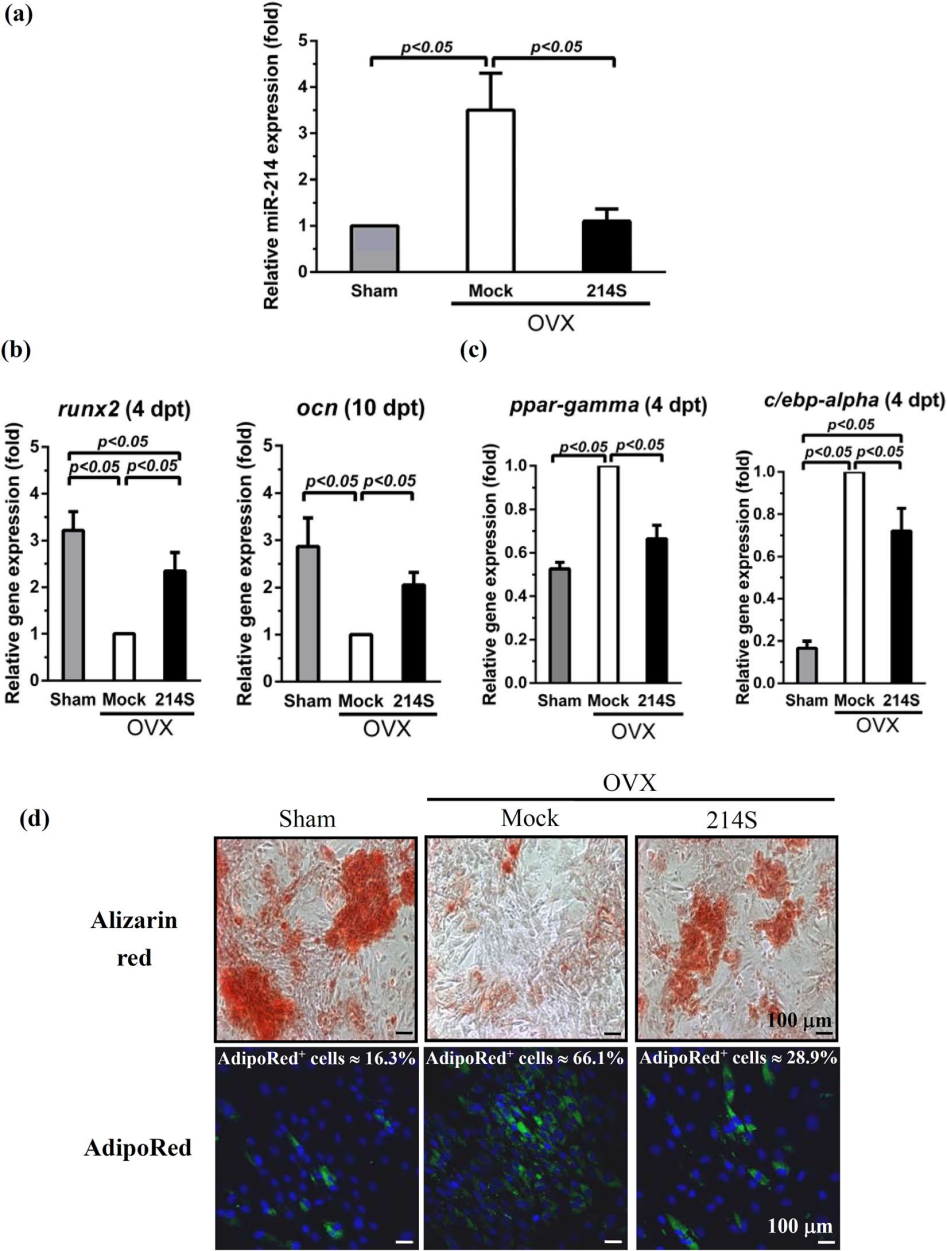
miR-214 regulates the switching of adipogenesis and osteogenesis in OVX-ASCs. OVX-ASCs were mock-transduced (Mock group) or co-transduced with BacECre/Bac214S at MOI 100/150 (214 S group). ASCs isolated from Sham animals (Sham) served as a control. (**a**) qRT-PCR analysis of miR-214. The cells were harvested at 2 days post-transduction (dpt) for analysis. (**b**) qRT-PCR analysis of marker genes in early (*runx2*) and middle (*ocn*) stage of osteogenic differentiation at 4 and 10 dpt, respectively. (**c**) qRT-PCR analysis of adipogenic marker genes (*ppar-γ* and *c/ebp-α*) at 4 dpt. (**d**) Alizarin red and AdipoRed staining performed at 14 dpt. Alizarin red stained calcium and hence mineralization. AdipoRed^TM^ stained intracellular lipid droplets and cells committing to adipogenic lineage emitted green fluorescence under the confocal microscope. The percentages of cells emitting fluorescence are shown.

To examine the miR-214 levels in osteoporotic ASCs, we first created osteoporotic rat models by ovariectomy (OVX). ASCs were isolated from animals with (OVX-ASCs) or without OVX (Sham-ASCs). OVX-ASCs were mock-transduced (transduced without BV, Mock group) or co-transduced with 2 recombinant BV BacECre (expressing Cre) and Bac214S (expressing 10 repeats of miR-214 sponge, Fig. S1), which prolongs miR-214 sponge expression for > 14 days and downregulates miR-214 in OVX-BMSCs^12^. qRT-PCR analysis (Fig. 1a) revealed ≈3.5 fold miR-214 expression in mock-transduced OVX-ASCs (Mock) as opposed to Sham-ASCs. Likewise, co-transduction of OVX-ASCs with BacECre/Bac214S (214 S group) knocked down the endogenous miR-214 to a level statistically similar (*p* > 0.05) to that in Sham-ASCs (Fig. 1a).

To explore whether OVX-ASCs favored adipogenic differentiation and the role of miR-214 overexpression, OVX-ASCs (Mock and 214 S groups) and Sham-ASCs (Sham group) were cultured in osteoinduction or adipoinduction medium and analyzed by qRT-PCR. In comparison with the Sham group, the Mock group expressed lower (*p* < 0.05) levels of osteogenic (*runx2* and *ocn*) genes upon osteoinduction (Fig. 1b) but higher (*p* < 0.05) levels of adipogenic (*ppar-γ* and *c/ebp-α*) genes upon adipoinduction (Fig. 1c). Alizarin red and AdipoRed staining performed at day 14 (Fig. 1d) further revealed that Mock group exhibited poorer mineralization (upper panel) and more triglycerides accumulation (lower panel) than Sham group. Conversely, 214 S group significantly (*p* < 0.05) elevated the *runx2* and *ocn* expression (Fig. 1b), attenuated the *ppar-γ* and *c/ebp-α* expression (Fig. 1c), triggered more evident mineralization and dampened the accumulation of intracellular triglycerides at 14 dpt (Fig. 1d). These data collectively confirmed that OVX-ASCs aberrantly overexpressed miR-214 and favorably committed to adipogenic rather than osteogenic lineage, but alleviating miR-214 level switched the differentiation from adipogenic towards osteogenic lineage.

### miR-214 targeted TAB2 and CTNNB1 in the Wnt pathway to regulate OVX-ASCs differentiation

**Figure 2:**
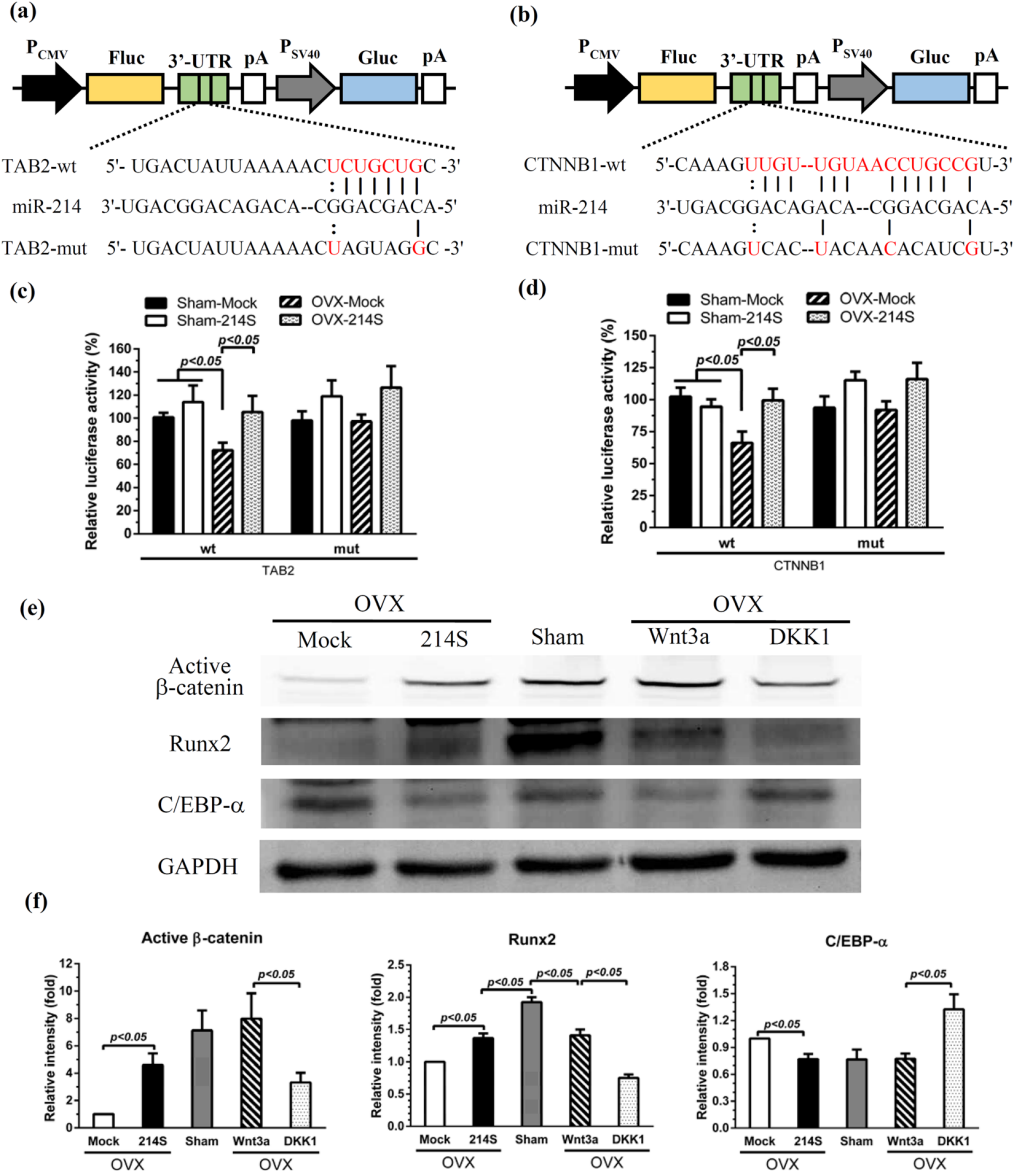
miR-214 targeted *TAB2* and *CTNNB1* in the Wnt pathway to switch osteogenesis/adipogenesis. (**a**) Reporter plasmids expressing Gluc and Fluc, with wild-type or mutant *TAB2* (TAB2-wt or TAB2-mut) sequences at the 3′ UTR of Fluc. (**b**) Reporter plasmids expressing Gluc and Fluc, with wild-type or mutant *CTNNB1* (CTNNB1-wt or CTNNB1-mut) sequences at the 3′ UTR of Fluc. (**c**) Relative luciferase activities in cells transfected with TAB2-wt or TAB2-mut. (**d**) Relative luciferase activities in cells transfected with CTNNB1-wt or CTNNB1-mut. (**e**) Western blot analysis. (**f**) Densitometry analysis of bands in Western blot. Sham-ASCs and OVX-ASCs were mock-transduced (Sham-Mock and OVX-Mock) or co-transduced with BacECre/Bac214S (Sham-214S and OVX-214S). Cells were transfected with one of these 4 plasmids, followed by measurement of Fluc and Gluc activities 3 days later. Transfection efficiency was calibrated by Gluc activity and Fluc/Gluc were normalized to those in the Sham-Mock or Sham-214S groups to yield relative luciferase activities.

To dissect how miR-214 regulated the adipogenesis/osteogenesis switching, we performed bioinformatic prediction, which revealed high complementarity between miR-214 and the 3′-UTR of *TAB2* and *CTNNB1* genes. Therefore we constructed 4 reporter plasmids expressing Gaussia luciferase (Gluc) and firefly luciferase (Fluc), with wild-type or mutant *TAB2* (TAB2-wt or TAB2-mut, Fig. 2a) or *CTNNB1* (CTNNB1-wt or CTNNB1-mut, Fig. 2b) sequences at the 3′-UTR of Fluc. If TAB2 or CTNNB1 are targets of miR-214, high levels of miR-214 can bind to TAB2-wt or CTNNB1-wt and suppress the Fluc expression, but high levels of miR-214 would not bind to TAB2-mut or CTNNB1-mut to repress Fluc expression.

Sham-ASCs and OVX-ASCs were mock-transduced (Sham-Mock and OVX-Mock) or co-transduced with BacECre/Bac214S (Sham-214S and OVX-214S), followed by transfection with one of these 4 plasmids and measurement of Fluc and Gluc activities 3 days later. Transfection efficiency was calibrated by Gluc activity and the relative luciferase activities were obtained by normalizing Fluc/Gluc to those in the Sham-Mock or Sham-214S groups.

Compared with the Sham-Mock (expressing low levels of miR-214) transfected with the same plasmids, transfection of OVX-Mock (expressing high levels of miR-214) with TAB2-wt (Fig. 2c) or CTNNB1-wt (Fig. 2d) significantly (*p* < 0.05) reduced the luciferase activity, but transfection of OVX-Mock and OVX-214S with TAB2-mut or CTNNB1-mut did not diminish the luciferase activity, indicating that miR-214 targeted *TAB2* and *CTNNB1* genes.

*TAB2* gene encodes TAB2 that transmits noncanonical Wnt signaling^20^ while *CTNNB1* encodes β-catenin which is a pivotal mediator in the canonical Wnt signaling to activate osteogenic transcription factor (TF) Runx2 and suppress adipogenic TF C/EBP-α. To elucidate whether miR-214 blocked the Wnt pathway, cells in the Mock, 214 S and Sham groups were analyzed at 3 dpt by Western blot (Fig. 2e) and densitometry (Fig. 2f). As controls, Sham-ASCs and OVX-ASCs were treated with the Wnt pathway activator (Wnt3a) or inhibitor (DKK1^21^) for 3 days. Compared with the Sham group, the Mock group (expressing abundant miR-214) expressed significantly less (*p* < 0.05) active β-catenin/Runx2, but more C/EBP-α. Nonetheless, reducing miR-214 levels in OVX-ASCs (214 S group) raised the levels of active β-catenin/Runx2 and repressed the C/EBP-α expression (Fig. 2e,f). Such osteoinductive and adipo-suppressive effects exerted by the 214 S group coincided with those by Wnt3a treatment (Wnt activation) but were contrary to those by DKK1 treatment (Wnt inhibition). Therefore, suppressing miR-214 regulated osteogenesis/adipogenesis via the Wnt pathway.

### Suppressing miR-214 in OVX-ASCs stimulated the osteogenesis of co-cultured OVX-BMSCs

**Figure 3:**
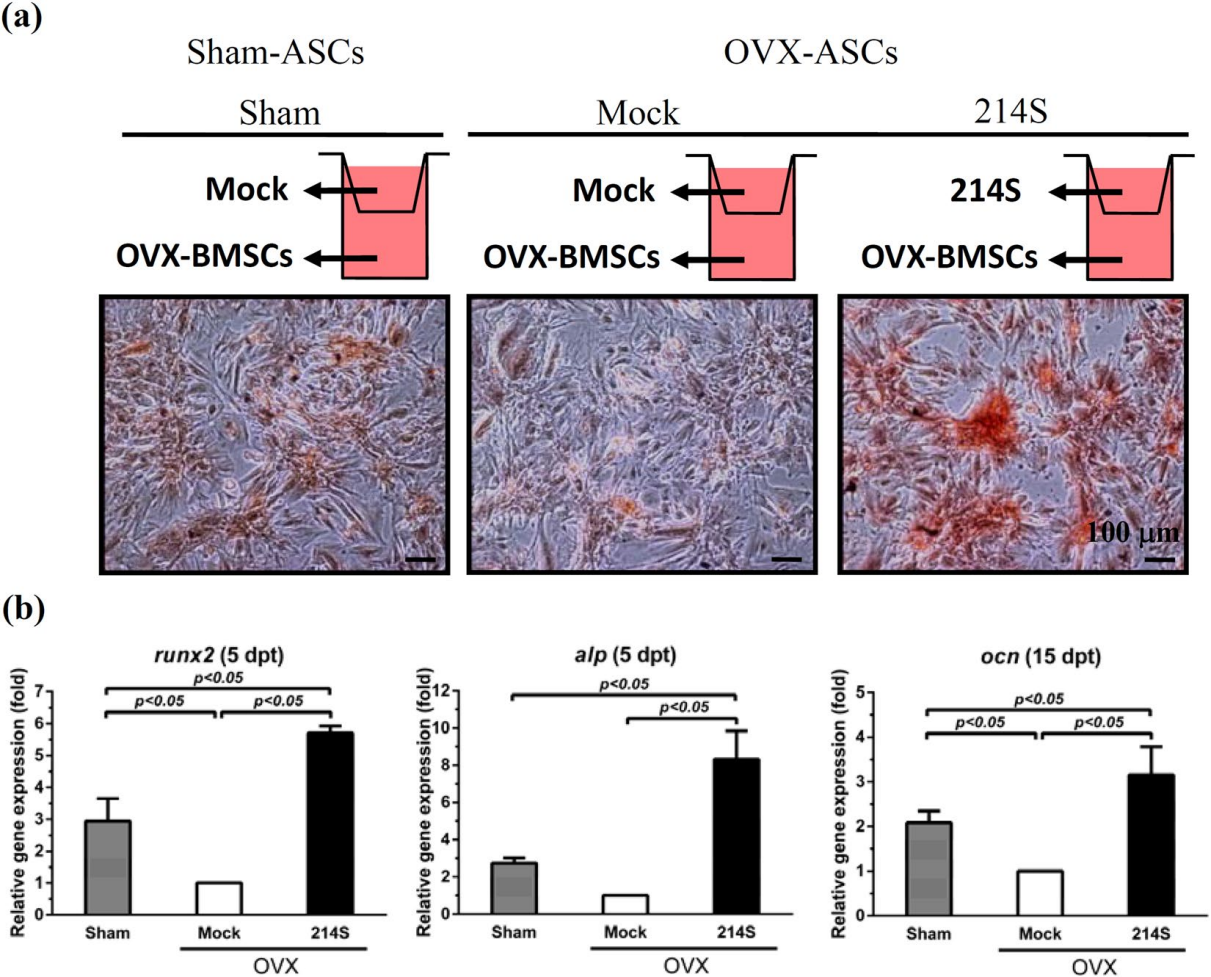
Suppressing miR-214 in OVX-ASCs stimulated the osteogenesis of co-cultured OVX-BMSCs. (**a**) Co-culture of ASCs (Sham or OVX) with OVX-BMSCs in transwell assays and Alizarin red staining. (**b**) qRT-PCR analysis of osteogenic genes. Sham-ASCs (Sham), mock-transduced OVX-ASCs (Mock) and BacECre/Bac214S-transduced OVX-ASCs (214S) were seeded to transwell inserts, while OVX-BMSCs were seeded to the bottom of transwell plates. The cells were co-cultured in osteogenic medium for 15 days. OVX-BMSCs were stained by Alizarin red at 15 dpt and analyzed by qRT-PCR for osteogenic gene expression.

Whether OVX-ASCs affected surrounding OVX-BMSCs was performed by co-culturing OVX-BMSCs with Sham-ASCs (Sham) or OVX-ASCs (Mock or 214 S group) for 15 days in the transwell plates (Fig. 3a). Compared with the Sham group, the Mock group attenuated the OVX-BMSCs osteogenesis as judged from poorer mineralization (Alizarin red staining, Fig. 3a), and significantly lower (*p* < 0.05) levels of *runx2*, *alp* and *ocn* in OVX-BMSCs (Fig. 3b). In marked contrast, the 214 S group triggered remarkably more evident mineralization (Fig. 3a) and higher levels of *runx2*, *alp* and *ocn* in OVX-BMSCs (Fig. 3b) than the Sham and Mock groups, indicating that BacECre/Bac-214S-transduced OVX-ASCs stimulated the OVX-BMSCs osteogenesis, via a paracrine fashion.

### Suppressing miR-214 in OVX-ASCs altered exosomal miR-214 and cytokine secretion

**Figure 4:**
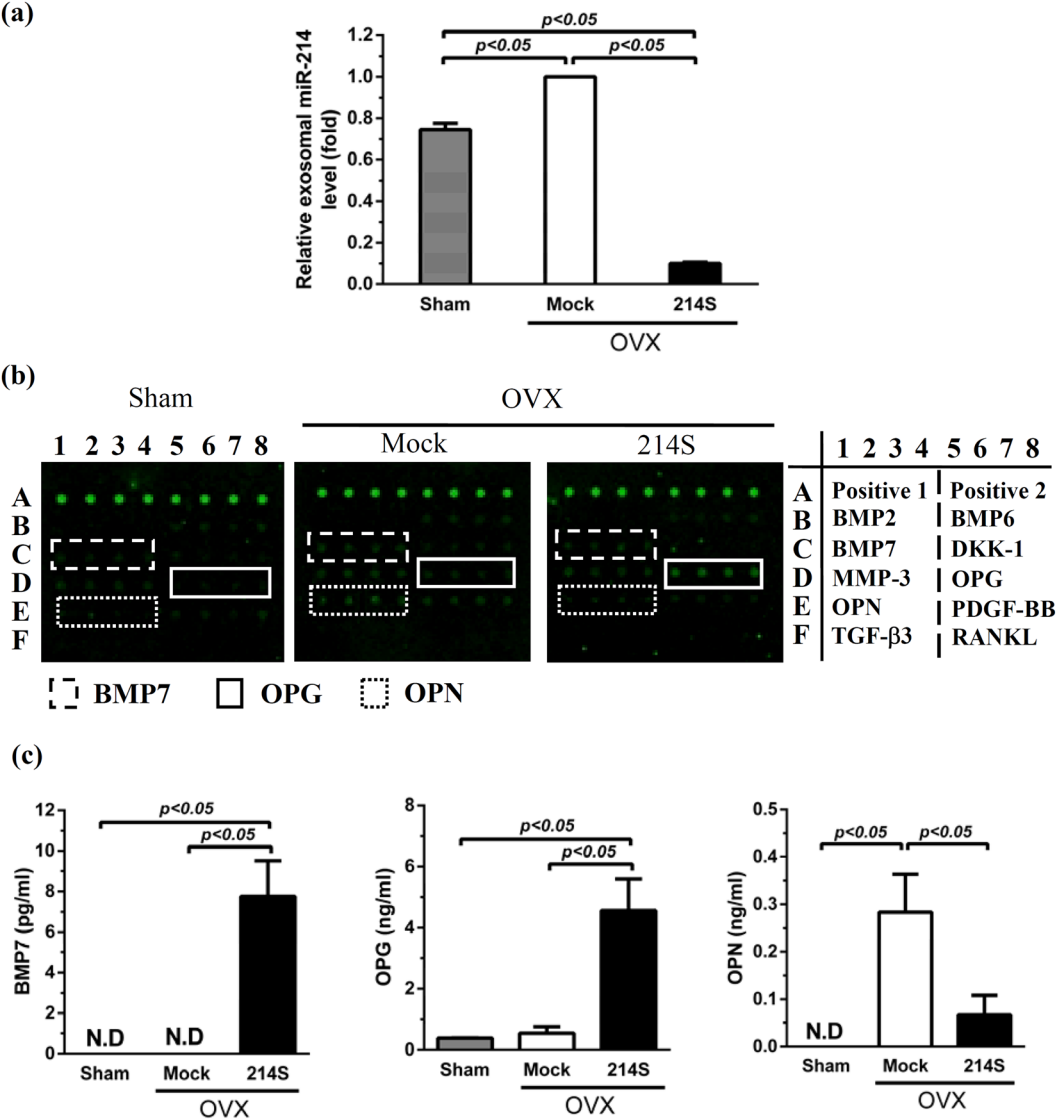
Suppressing miR-214 in OVX-ASCs altered exosomal miR-214 and cytokine secretion. (**a**) Exosomal miR-214 level relative to that of Mock group. (**b**) Protein assay images. (**c**) Quantitative protein array data. We mock-transduced OVX-ASCs (Mock group) or co-transduced OVX-ASCs (214S group) as in Fig. 1, isolated extracellular exosomes at 3 dpt and analyzed the exosomal miR-214 by qRT-PCR. Exosomal miR-214 from Sham-ASCs were analyzed in the same manner. Cytokines in the supernatant were analyzed at day 15 using a protein array.

We next explored what were secreted from the OVX-ASCs. BMSCs can release exosomes that are vesicles carrying functional RNA (e.g. miRNA) for paracrine signaling between BMSCs and nearby cells^22^. Therefore, we mock-transduced OVX-ASCs (Mock group) or co-transduced OVX-ASCs (214 S group) as in Fig. 1, isolated exosomes at 3 dpt from the supernatant and analyzed the exosomal miR-214 by qRT-PCR. Exosomal miR-214 from Sham-ASCs were analyzed in the same manner. Figure 4a delineates that the Mock group (highly expressed miR-214) secreted more (*p* < 0.05) exosomal miR-214 than the Sham-group. However, the 214 S group released significantly lower levels of exosomal miR-214, indicating that suppressing intracellular miR-214 concomitantly repressed the secretion of exosomal miR-214.

Furthermore, we analyzed 10 bone-associated cytokines in the supernatant at day 15 using a protein array. The qualitative (Fig. 4b) and quantitative (Fig. 4c) array data revealed that 214 S group secreted significantly higher levels of BMP7 and osteoprotegerin (OPG) and lower levels of osteopontin (OPN) than the Mock group.

### Regeneration of osteoporotic bone defects by the engineered OVX-ASCs: μ*CT evaluation*

To explore the potential of BV-engineered OVX-ASCs for osteoporotic bone defect healing, we created osteoporotic rat models and drilled critical-size defects (3 mm in diameter) at the left femoral metaphysis. OVX-ASCs were mock-transduced (Mock group), co-transduced with BacECre/BacLEBW (LEBW group, for sustained BMP2 expression, Fig. S1 and ref.^12^), BacECre/Bac214S (214 S group) or BacECre/BacLEBW/Bac214S (LEBW/214 S group for sustained BMP2/miR-214 sponge expression), loaded into gelatin scaffolds at 1 dpt, and implanted into the defects (*n* = 8 for each group at week 5 (5W); *n* = 4 for each group at week 2 (2W)).

**Figure 5:**
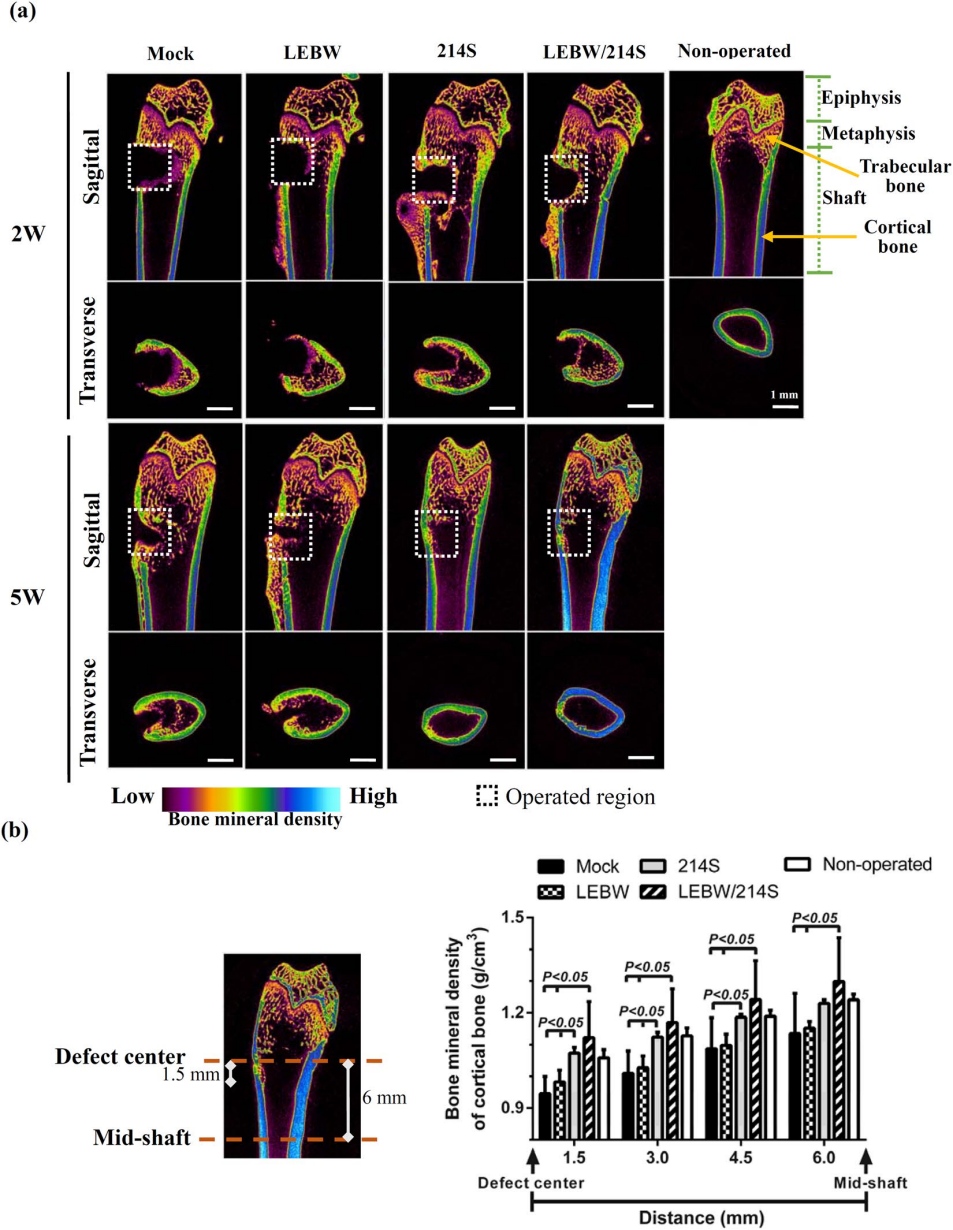
Bone healing as evaluated by μCT. OVX-ASCs were mock-transduced (Mock group), co-transduced with BacECre/BacLEBW (LEBW group for BMP2 expression), BacECre/Bac214S (214S group for miR-214 sponge expression) or BacECre/BacLEBW/Bac214S (LEBW/214S group for sustained BMP2/miR-214 sponge expression), loaded into gelatin scaffolds at 1 dpt, and implanted into the critical-size defects (3 mm in diameter) at the left femoral metaphysis of osteoporotic rats. (**a**) μCT images at weeks 2 (2W) and 5 (5 W). *n* = 8 for each group at 5 W; *n* = 4 for each group at 2 W. The defect areas are indicated by dashed box. The color intensity indicates bone density. (**b**) BMD of cortical bone from defect center to mid-shaft of the LEBW/214S group.

The μCT imaging at 2W and 5W illustrated poor bone healing and shattered bone structure near the implantation site in the Mock and LEBW groups (Fig. 5a), indicating that implanting OVX-ASCs and even OVX-ASCs expressing potent osteogenic factor BMP2 failed to heal the defects. Intriguingly, the cortical bone in the Mock and LEBW groups at 5W were also compromised and the bone mineral densities (BMD) were evidently lower than those of the intact cortical bone. Conversely, both the 214 S and LEBW/214S groups completely filled the defects with osseous tissues at 5W (Fig. 5a). Of note, at 5W the LEBW/214S group not only increased the BMD at the defect (transverse view, Fig. 5a), but also elevated the cortical bone BMD along the shaft (sagittal view, Fig. 5a). Quantitative μCT analysis (Fig. 5b) attested that the BMD from defect center to the mid-shaft in the LEBW/214S group significantly (*p* < 0.05) exceeded those in the Mock and LEBW groups, and was superior to those in the 214S group and non-operated bone although the difference was statistically insignificant.

**Figure 6:**
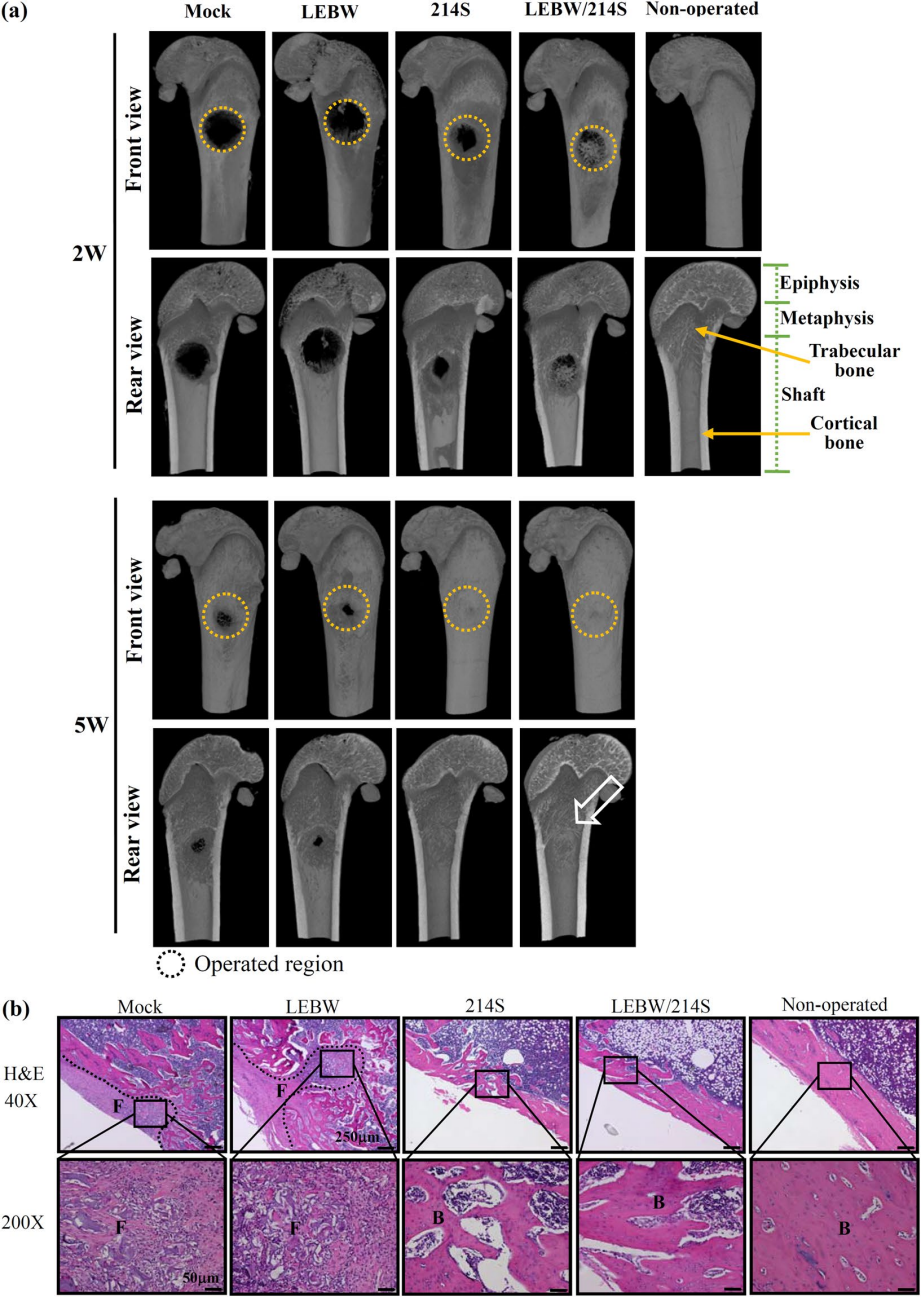
μCT and histological analyses. (**a**) Front and rear views of reconstructed 3D μCT images. Empty triangle at 5 W points to the trabecular bone. (**b**) H&E staining.

The front and rear views of 3D images (Fig. 6a) further confirmed that the Mock and LEBW groups failed to completely fill the defect at 2W and 5W, whereas the 214S and LEBW/214S groups filled the entire defects in both exterior and interior sides at 5W. Of note, the LEBW/214S group also improved trabecular bone-like structure formation within the metaphysis at 5W. After μCT scanning at 5W, the left femora were removed for analyses. The Mock and LEBW groups were filled with fibrous tissues within the defect areas as judged from the H&E staining (Fig. 6b). The 214S group improved the bone formation while the LEBW/214S group yielded even better new bone formation with abundant matrix and more compact bone structure, which histologically resembled the non-operated bone (Fig. 6b).

**Figure 7:**
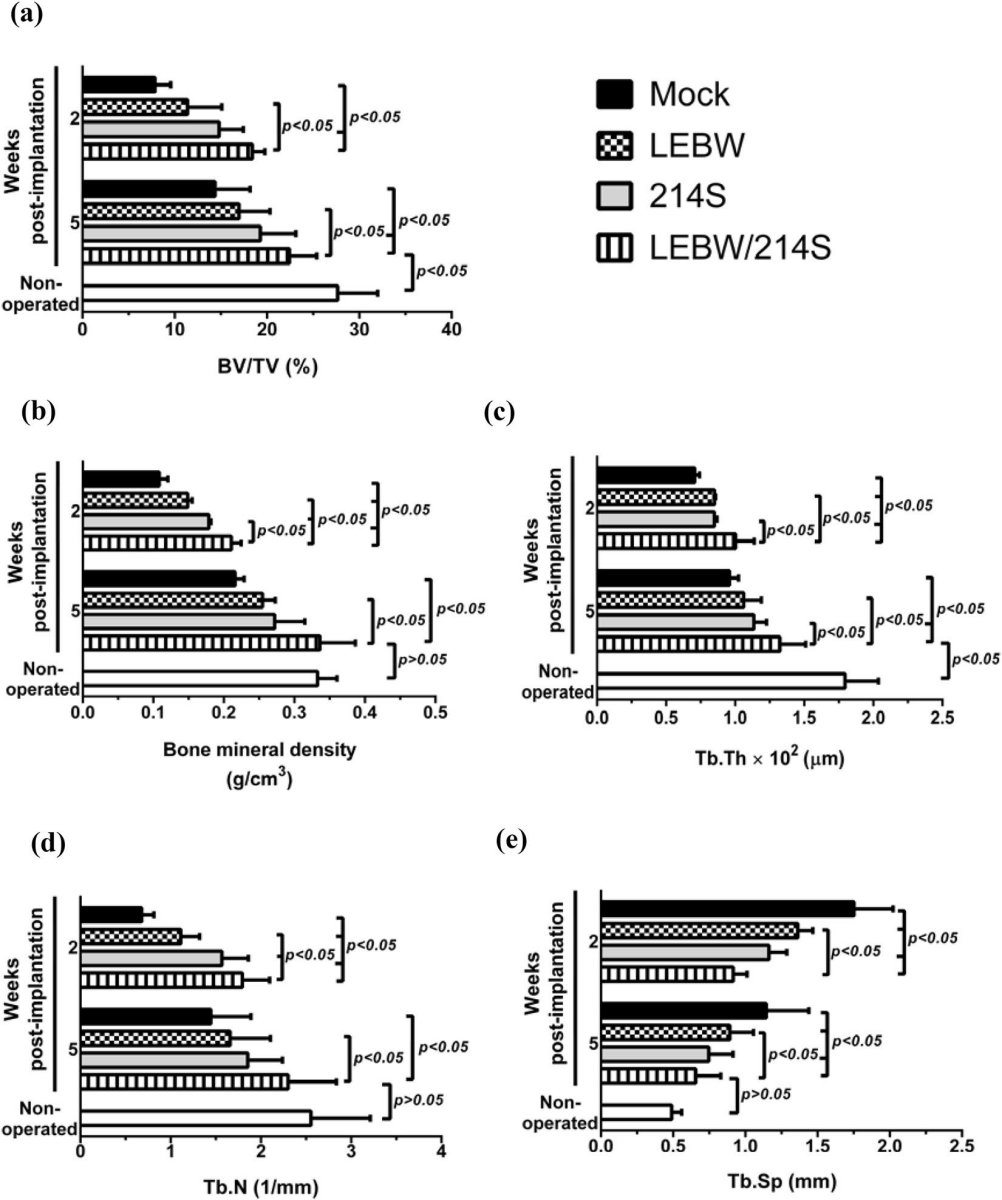
Bone formation and microarchitecture parameters analyzed using μCT imaging data. (**a**) Bone volume/total volume (BV/TV). (**b**) Bone mineral density. (**c**) Tb.Th (average trabecular thickness). (**d**) Tb.N (trabecular numbers/mm). (**e**) Tb.Sp (average distance between trabeculae).

Furthermore, we calculated bone formation and microarchitecture parameters in the operated region using the μCT data. Compared with the Mock and LEBW groups, the 214S group gave rise to higher bone volume to total volume ratio (BV/TV, Fig. 7a), BMD (Fig. 7b), trabecular thickness (Tb. Th, Fig. 7c) and trabecular number (Tb.N, Fig. 7d) as well as lower distance between trabeculae (Tb.Sp, Fig. 7e) at 5W, although the difference was not statistically significant in all parameters. Conversely, at 5W the LEBW/214S group conferred significantly (*p* < 0.05) higher BV/TV, BMD, Tb.Th, Tb.N and lower Tb.Sp than the Mock and LEBW groups, and had statistically similar (*p* > 0.05) BMD, Tb.N and Tb.Sp when compared with the non-operated group. These data altogether demonstrated that OVX-ASCs co-expressing BMP2 and miR-214 sponges revived the bone microarchitecture in osteoporotic rats.

## Discussion

The overriding objective of this study was exploiting osteoporotic ASCs to heal bone defects in osteoporotic rats. Using OVX rats as the osteoporotic animal model, we found that OVX-ASCs highly expressed miR-214 and exhibited impaired osteogenesis capability but favored adipogenic differentiation (Fig. 1). Such finding agreed with the observations in osteoporotic BMSCs whose lineage commitment favorably shift to adipogenesis^23–25^. Nonetheless, by using the hybrid BV persistently expressing miR-214 sponge (214S group), we were able to knockdown miR-214 in OVX-ASCs to a level similar to that in Sham-ASCs, hence reversed the adipogenic/osteogenic differentiation bias and enhanced OVX-ASCs osteogenesis (Fig. 1).

miR-214 was recently found to target baculoviral IAP repeat-containing 7 in human osteoblasts^26^, Osx in C2C2 cells^27^ and ATF4 (another TF important for osteogenic differentiation) in mouse OVX-BMSCs^28^. Here we unraveled two new miR-214 targets: *CTNNB1* (β-catenin) and *TAB2* (Fig. 2). β-catenin and TAB2 are key mediators in the Wnt pathway^21,29^ which stimulates Runx2 expression and directs mesenchymal precursor commitment to osteogenic lineage^30,31^ while simultaneously represses chondrogenesis^32^ and adipogenesis^33,34^. In the canonical Wnt pathway, β-catenin promotes BMSCs osteogenesis through its interaction with Runx2 to augment bone formation^35,36^ while prevents BMSCs adipogenesis by downregulating PPAR-γ and C/EBP-α^7,37^. In the noncanonical Wnt pathway, TAB2 is a scaffold protein responsible for transmitting the signal^37^. Consequently, miR-214 overexpression in OVX-ASCs suppressed β-catenin and TAB2, resulting in the blockade of Wnt signaling and impaired osteogenesis potential. However, suppressing miR-214 by the BV-expressed miR-214 sponge reprogrammed the differentiation preference. These data shed light on how miR-214 functions as a “molecular switch” controlling the OVX-ASCs differentiation, and provided new insights into why repressing miR-214 levels can shift the OVX-ASCs differentiation from adipogenic to osteogenic.

Additionally, we uncovered that knocking down miR-214 levels in OVX-ASCs (214S group) stimulated the osteogenesis of co-cultured OVX-BMSCs via a paracrine manner (Fig. 3), which was accompanied by reduced release of exosomal miR-214 and OPN as well as increased secretion of BMP7 and OPG (Fig. 4). Very recently it was reported that exosomal miR-214 secreted by osteoclast can be transferred to osteoblasts to inhibit osteoblastic activity^38^. Conversely, BMP7 can promote osteogenic differentiation of osteoporotic BMSCs^25^. The alleviated exosomal miR-214-triggered inhibition and enhanced BMP7-mediated stimulation contributed to the enhanced osteogenesis of co-cultured OVX-BMSCs. The OPN and OPG might be implicated in the *in vivo* repair (see below).

Owing to the compromised osteogenesis capability of OVX-ASCs, implantation of the mock-transduced OVX-ASCs (Mock group) failed to heal the bone defects in the OVX rats (Figs 5–7). Even engineered OVX-ASCs persistently expressing the potent osteoinductive BMP-2 (LEBW group) failed to heal the defects (Figs 5–7), which underlined the difficulty to heal the bone defects in osteoporotic patients/animals using autologous cells. This also suggested that in OVX-ASCs either BMP2 is insufficient to activate the downstream BMP and MAPK/ERK pathways, or these pathways require additional cues to orchestrate osteogenic differentiation. The latter conjecture is supported by our recent finding that implantation of rat ASCs co-expressing BMP2 and SDF1 (which potentiates MAPK/ERK pathway and stabilizes Runx2) repairs difficult-to-heal calvarial bone defects more efficiently than ASCs expressing BMP2 alone^39^.

Despite the difficulty to repair osteoporotic bone defects, implanting OVX-ASCs whose intracellular miR-214 level was knocked down by BV-expressed miR-214 sponge (214S group) remarkably repaired the bone defects in 5 weeks (Figs 5–7). Such striking healing may be ascribed to several factors. First, suppressing miR-214 reprogramed the propensity of OVX-ASCs differentiation from adipogenic to osteogenic by reviving the Wnt signaling mediator β-actin/TAB2 (Figs 1 and 2). Second, implantation surgery itself provokes host BMSCs infiltration to the injury site^40^ and the implanted OVX-ASCs stimulated the osteogenesis of host OVX-BMSCs (Fig. 3) by secreting osteoinductive BMP7 (Fig. 4). Third, exosomal miR-214 undermines *in vivo* bone formation^38^. Suppressing intracellular miR-214 in OVX-ASCs concomitantly decreased the exosomal miR-214 secretion, thereby alleviating the inhibitory effects and promoting bone repair. Fourth, OPG is an antagonist of RANKL that inhibits osteoclast maturation. OVX-ASCs transduced with BacECre/Bac214S secreted more OPG (Fig. 4), which can repress the maturation of osteoclast that impedes bone formation early in the regeneration process. In the later stage of repair process, the implanted cells should have been eradicated^41^, so that at 5W no OPG was secreted from the implanted cells to inhibit osteoclast.

By co-expressing BMP2 and miR214 sponge in the OVX-ASCs, the LEBW/214S group further substantiated the bone regeneration, as evidenced by the complete defect filling, improved cortical bone structure (Figs 4 and 5), highest BV/TV, BMD, Tb.Th, Tb.n and lowest Tb.Sp among all groups (Fig. 7). These data demonstrated that co-expressing BMP2/miR-214 sponge in OVX-ASCs exerted synergistic bone healing effects when compared with expressing miR-214 sponge alone. Since β-catenin pathway enhances mesenchymal cell responsiveness to BMP2^35^ and β-catenin requires interactions with such stimulatory signals as BMP2 to induce osteogenesis of stem cells^36^, it is likely that BMP2 not only coordinated with BMP7 to trigger BMP and MAPK/ERK signaling cascades but also potentiated the Wnt/β-catenin pathway, which acted in concert to provoke the osteogenesis and suppress adipogenesis of OVX-ASCs, thereby further ameliorating the bone repair. Furthermore, miR-214 and miR-148b antagonize the effects of each other in cancer cells^42^. miR-148b can effectively enhance ASCs osteogenesis^43^ by targeting noggin^44^, a BMP2 antagonist that negatively regulates BMP2-induced osteoblast differentiation and bone formation^3^. It is possible that suppressing miR-214 concurrently elevates miR-148b and decreases noggin expression, thus enhancing bone repair by alleviating the negative regulation. It should also be noted that implantation surgery induces host immune responses that trigger death of implanted cells. We previously found that implantation of rabbit ASCs into the femoral defects in rabbits healed the critical-size defects but the implanted cells were eradicated in 4 weeks^40^. Since the cells were implanted into immunocompetent rats, it was likely that the cells were eradicated after a period of time. However, the fate of implemented cells awaits further investigation.

**Figure 8:**
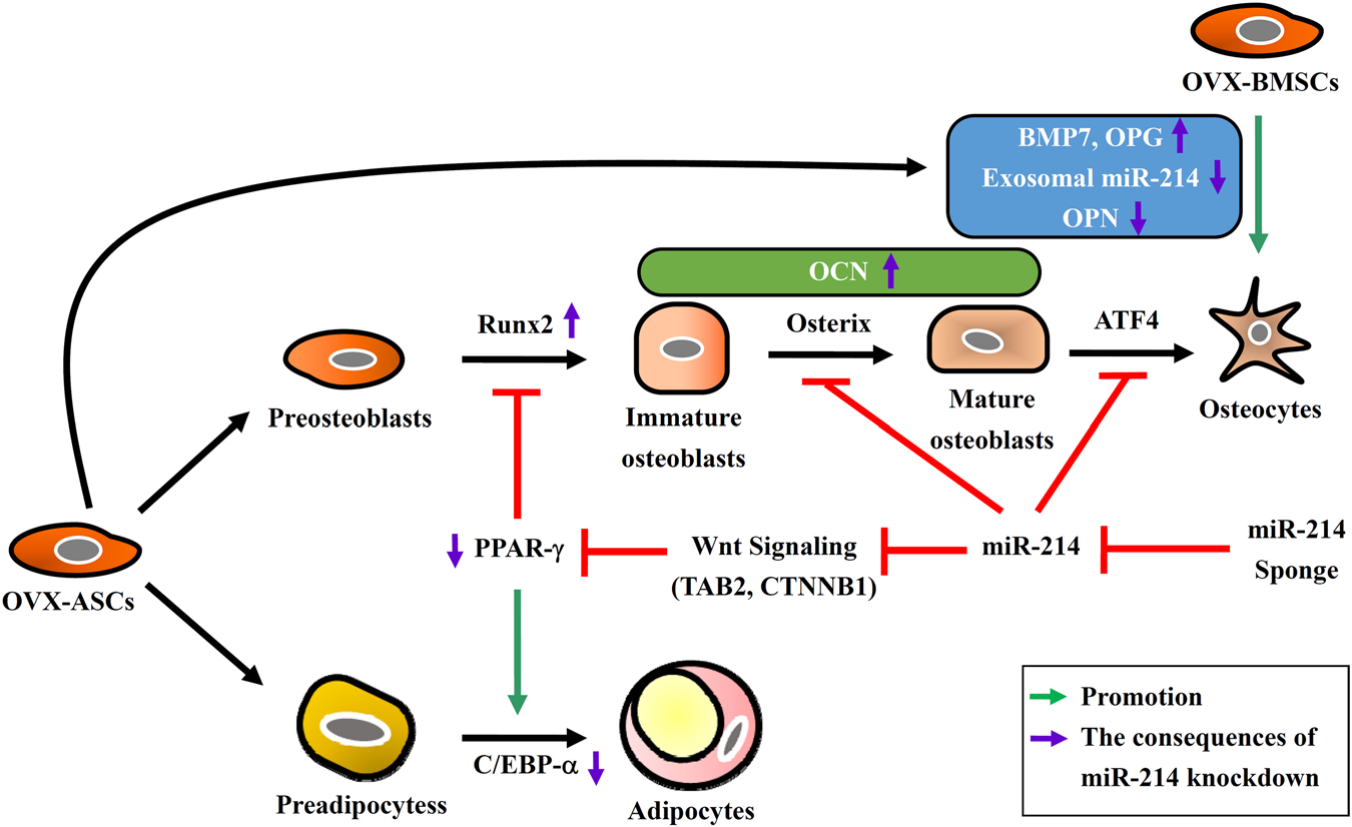
A model accounting for how miR-214 orchestrates osteogenesis and adipogenesis in OVX-ASCs.

In light of our data, we propose a model (Fig. 8) that OVX-ASCs express aberrantly high level of miR-214, which targets *TAB2* and *CTNNB1* and hence blocks the Wnt pathway, leading to the overexpression of PPAR-γ and C/EBP-α and preferential differentiation into adipocytes. miR-214 also targets osterix^27^ and ATF4^28^ that dictate the differentiation into mature osteoblasts and to osteocytes, respectively, thereby further repressing osteogenesis. High miR-214 levels in OVX-ASCs also increases exosomal miR-214/OPN secretion but decreases BMP7/OPG secretion, which concurs with compromised osteogenesis of co-cultured OVX-BMSC. Consequently, suppressing miR-214 activates Wnt pathway, which not only blocks PPAR-γ/CEBP-α to mitigate adipogenesis but also enhances Runx2 levels, de-represses osterix and ATF4, hence additively promoting the osteogenic differentiation of OVX-ASCs. Meanwhile, knocking down miR-214 levels in OVX-ASCs enhances BMP7/OPG secretion and lowers exosomal miR-214/OPN secretion, which simultaneously enhances the osteogenesis of host OVX-BMSCs and attenuates the host osteoclast activity. These mechanisms converge to improve the commitment of miR-214 sponge-expressing OVX-ASCs towards osteogenic lineage and augment bone healing in OVX rats via autocrine and paracrine effects. BMP2/miR-214 sponge co-expression further synergizes the OVX-ASCs osteogenesis and bone healing in OVX rats. In conclusion, this study implicates the potential of engineering osteoporotic ASCs by repressing miR-214 as a means to treat osteoporotic fractures.

## Methods

### Osteoporotic rat model, ASCs and BMSCs isolation and expansion

All animal experiments were performed in compliance with the Guide for the Care and Use of Laboratory Animals (Ministry of Science and Technology, Taiwan) and experimental protocols were approved by the Institutional Animal Care and Use Committee of National Tsing Hua University. To generate osteoporotic rat models, Sprague-Dawley female rats (8-weeks old, BioLASCO, Taiwan) were subjected to bilateral ovariectomy (OVX) or sham operation (Sham) as described^12^. After ovariectomy, the rats were injected with methylpredinisolone hemisuccinate (1 mg/kg body weight/day, Sigma) every day for 4 weeks. Osteoporosis induced by this method was confirmed previously^12^.

ASCs were harvested subcutaneously from the inguinal fat pads of OVX rats (OVX-ASCs) or Sham rats (Sham-ASCs) and isolated the same way as porcine ASCs isolation^45^. BMSCs were isolated from the bone marrow of hind limb of OVX rats (OVX-BMSCs) or Sham rats (Sham-BMSCs) as described^12^. All isolated cells were cultured in DMEM medium containing 10% fetal bovine serum (FBS, Hyclone), 100 IU/ml penicillin and 100 IU/ml streptomycin, incubated at 37 °C (5% CO_2_) and were passaged 3–5 times for experiments.

### Recombinant BV preparation and transduction

All BV vectors were constructed previously^12^, amplified by infecting Sf-9 cells and tittered as described^19^. BacECre transiently expressed Cre recombinase; BacLEBW expressed human bone morphogenetic protein 2 (BMP2) and Bac214S expressed d2EGFP with 10 tandem hsa-miR-214-3p binding sites (miR-214 sponge) at the 3′-UTR. The expression cassettes in hybrid substrate vectors BacLEBW and Bac214S were flanked by loxP sites for Cre recognition, excision and recombination (Fig. S1).

OVX-ASCs were transduced with BV vectors as described^19^ and in Supplementary Methods. After 6 h of transduction, the cells were cultured in osteoinduction medium (DMEM containing 10% FBS, 100 IU/ml penicillin, 100 IU/ml streptomycin, 0.1 μM dexamethasone, 10 μM β-glycerol phosphate and 50 μM ascorbic acid 2-phosphate) or adipoinduction medium (Hyclone) containing 3 μM sodium butyrate (Sigma). After 15 h of incubation at 37 °C, the medium was removed and cells continued to be cultured using fresh osteoinduction or adipoinduction medium.

### Quantitative real-time reverse-transcription PCR (qRT-PCR) and miR-214 analysis

Total cellular RNA was isolated using the NucleoSpin RNA II kit (Machereye-Nagel) and reverse transcribed to cDNA using the Omniscript RT Kit (Qiagen). The osteogenic and adipogenic genes were analyzed using StepOnePlus Real-Time PCR Systems (Applied Biosystems) and gene-specific primers (Table S1). Mature miRNAs in the cells were isolated using Trizol (Invitrogen) while miR-214 in the exosomes were isolated using miRCURY™ Exosome Isolation Kit (Exiqon), followed by miRNA extraction using Trizol. The cellular and exosomal miR-214 levels were analyzed using the TaqMan MicroRNA Assays kit (Applied Biosystems). The gene expression levels were normalized against that of U6 (for miRNAs) or *gapdh* (for osteogenic and adipogenic genes) and referenced to those of selected cells (see Results).

### Alizarin red staining and AdipoRed^TM^ staining

Cells were stained by Alizarin red to assess mineralization as described^12^. To assess adipogenesis, cells were washed with PBS twice, stained 15 min using AdipoRed^TM^ (Lonza) which stains intracellular lipid droplets, and observed under a confocal microscope (TE2000-E, Nikon). The stained triglyceride emitted green fluorescence at 572 nm.

### Luciferase reporter assay

pCDNA3.1-dualLuc encoding firefly luciferase (Fluc) and Gaussia luciferase (Gluc) was previously constructed^12^. The potential miR-214-3p binding sequences at the 3′-UTR of *TAB2* and *CTNNB1* were predicted using the TargetScan 6.2 and microRNA.org database. The wild-type and mutant 3′-UTR sequences of *TAB2* and *CTNNB1* within the predicted target sites were chemically synthesized (Table S2) and separately cloned downstream of *fluc* to yield TAB2-wt, TAB2-mut, CTNNB1-wt or CTNNB1-mut plasmids.

For luciferase reporter assays, the 4 plasmids (2.5 μg) were separately transfected into cells using Lipofectamine 3000 (Invitrogen) and cells were collected 2 days later. The Fluc and Gluc activities in the lysates were measured with the Pierce™ Gaussia-Firefly Luciferase Dual Assay Kit (Thermo Fisher) and read using a liquid scintillation microplate reader (Spectra Max M2, Molecular Devices). Transfection efficiency was calibrated by Gluc activity and the relative luciferase activities were obtained by normalizing the ratio of Fluc to Gluc (Fluc/Gluc) to those in reference cells (see Results).

### Western blot

OVX-ASCs were (i) mock-transduced, (ii) co-transduced with BacECre/Bac214S; or treated with (iii) 100 ng/ml recombinant mouse Wnt3a (R&D Systems) or (iv) 100 ng/ml recombinant mouse DKK1 (R&D Systems) for 3 days. As a control, Sham-ASCs were cultured for 3 days. Proteins in cell lysates were subjected to Western blot as described in Supplementary Methods.

### Osteoinduction of OVX-BMSCs by transwell co-culture assay

The transwell co-culture assay was performed using the 24-well transwell plates (Corning). The BacECre/Bac214S-transduced OVX-ASCs or mock-transduced ASCs (OVX or Sham) were seeded to the inserts (0.4 μm pore diameter) at 5 × 10^3^ cells/well while OVX-BMSCs were seeded to the bottom of the plates (5 × 10^3^ cells/well). The cells were co-cultured using the osteoinduction medium. At day 5, OVX-BMSCs were harvested for qRT-PCR analysis of *runx2* and *alp* expression. At day 15, OVX-BMSCs were harvested for qRT-PCR analysis of *ocn* expression or stained with Alizarin red.

### Protein array

For protein array analysis, the medium of transduced OVX-ASCs and mock-transduced ASCs (OVX or Sham) was collected at 15 dpt and analyzed using the Quantibody® Human Bone Metabolism Array (RayBiotech). The array was scanned and the data were analyzed using the Q-Analyzer software (RayBiotech).

### Implantation of ASCs/gelatin constructs into the femoral metaphysis defects in OVX rats

To prepare the scaffolds, the Spongostan™ gelatin sponge (porosity ≈ 97%, cat#MS0003, Ethicon) was cut into disks (diameter ≈ 3 mm) and immersed into saline solution (thickness ≈ 2 mm after immersion). The OVX-ASCs cultured in T-75 flasks were mock-transduced or transduced, trypsinized, resuspended in DMEM medium, evenly seeded into the gelatin sponge scaffolds (2 × 10^6^ cells/scaffold) in the 12-well plate. After 1 h, the ASCs/gelatin constructs were cultured in fresh medium containing 3 mM sodium butyrate for 12 h.

Meanwhile, the osteoporotic rat models were generated and anesthetized as described in Sec. 2.1. The distal epiphysis of the left femur was osteotomized using a 3-mm diameter trephine bur. The bone defect was implanted with 1 ASCs/gelatin construct (2 × 10^6^ cells/animal), rinsed with 0.9% saline and the deep muscle layer and skin were closed.

### μCT

At 2 and 5 weeks post-implantation, the rats were sacrificed. The femora were removed and scanned using an animal μCT imaging system (SkyScan 1174, Bruker, Belgium) and the 3D images were reconstructed as described^12^. Volume of interest (VOI, diameter = 3 mm) was drawn within the femoral metaphysis defect and the new bone volume over total volume (BV/TV), bone mineral density (BMD), trabecular thickness (Tb.Th), trabecular number (Tb.N) and distance between trabeculae (Tb.Sp) were calculated using the CTAn software^12^.

### Histological and immunohistochemical staining

After μCT scanning, the femora were decalcified, dehydrated, embedded and sectioned (thickness = 3 mm) from the femoral mid-shaft to distal epiphysis encompassing the defect site. The sections in the defect sites were subjected to hematoxylin and eosin (H&E) staining as described in Supplementary Methods.

### Statistical analysis

All quantitative data were analyzed using one-way analysis of variance (ANOVA) or student’s t-test using a two-tailed distribution. The *in vitro* data represent the means±SD of at least 3 independent culture experiments. p values smaller than 0.05 were considered significant.

## Electronic supplementary material

Supplementary data
