# Supplementary material for: Baculovirus-Mediated miR-214 Knockdown Shifts Osteoporotic ASCs Differentiation and Improves Osteoporotic Bone Defects Repair
Source: Sci Rep. 2017 Nov 24;7:16225. doi: 10.1038/s41598-017-16547-3 (PMC5701180; doi:10.1038/s41598-017-16547-3)
Supplement: Supplementary file 1 — Supplementary data [file 41598_2017_16547_MOESM1_ESM.doc]

**Baculovirus-Mediated miR-214 Knockdown Shifts Osteoporotic ASCs Differentiation and Improves Osteoporotic Bone Defects Repair**

Kuei-Chang Li^a, §^, Yu-Han Chang^b, c, §^, Mu-Nung Hsu^a^, Shih-Chun Lo^a^, Wan-Hua Li^a^ and Yu-Chen Hu^a, *^

^a^ Department of Chemical Engineering, National Tsing Hua University, Hsinchu, Taiwan, 300

^b^ Center for Tissue Engineering, Chang Gung Memorial Hospital, Taoyuan, Taiwan 333

^c^ Department of Orthopaedic, Chang Gung Memorial Hospital, Taoyuan, Taiwan, 333

Running Title: Suppressing miR-214 for osteoporotic bone healing

§ These two authors contributed equally to this work

*Corresponding Author

Phone: (886)3-571-8245

FAX: (886)3-571-5408

Email: yuchen@che.nthu.edu.tw

**Supporting Information**

**Supplementary Methods**

**Transduction of OVX-ASCs**

OVX-ASCs were seeded to 6-well plates (2×10^5^ cells/well) or T-75 flasks (5×10^6^ cells/flask), cultured overnight and washed twice with phosphate-buffered saline (PBS, pH 7.4). Depending on the multiplicity of infection (MOI), a certain volume of virus supernatant was mixed with NaHCO_3_-free DMEM at a volumetric ratio of 1:4 (total volume was 0.5 and 2 ml in 6-well plates and T-75 flasks, respectively) and added to the cells. Mock transduction was performed similarly except that virus-free TNM-FH medium was mixed with NaHCO_3_-free DMEM at a volumetric ratio of 1:4. The cells were gently shaken on a rocking plate at room temperature for 6 h. After 6 h, the virus mixture was replaced with osteoinduction medium (DMEM containing 10% FBS, 100 IU/ml penicillin, 100 IU/ml streptomycin, 0.1 μM dexamethasone, 10 μM β-glycerol phosphate and 50 μM ascorbic acid 2-phosphate) or adipoinduction medium (Hyclone) containing 3 μM sodium butyrate (Sigma). After 15 h of incubation at 37°C, the medium was removed and cells continued to be cultured using fresh osteoinduction or adipoinduction medium.

Western blot

The OVX-ASCs were (i) mock-transduced, (ii) co-transduced with BacECre/Bac214S; or treated with (iii) 100 ng/ml recombinant mouse Wnt3a (R&D Systems) or (iv) 100 ng/ml recombinant mouse DKK1 (R&D Systems) for 3 days. As a control, Sham-ASCs were cultured for 3 days. These cells were lysed using RIPA buffer (Millipore) and the proteins in the cell lysates were subjected to SDS-PAGE (10-12%) separation, followed by Western blot using rabbit anti-rat MAb specific for active β-catenin, Runx2, C/EBP-α or GAPDH (all from Cell Signaling) as the primary antibody. The secondary antibody was goat anti-rabbit HRP-conjugated IgG (Antibodies Incorporated). Proteins were detected with enhanced chemiluminescence (ECL) reagents (Pierce) and images were developed using a GeneGnome HR scanner (Syngene), followed by densitometry analysis using GeneTools software.

Histological staining

After μCT scanning, the femora were completely decalcified by immersing in 0.5 M EDTA (pH 8.0) for 15-20 days, and dehydrated in a series of graded concentration of ethanol from 70% to 100%. The specimen were embedded in paraffin and subjected to coronal sectioning (thickness=3 mm) from the femoral mid-shaft to distal epiphysis encompassing the defect site. The sections in the defect sites were stained with hematoxylin and eosin (H&E)

**Table S1. Sequences of primers used for qRT-PCR**.

| Genes | Forward primers | Reverse primers |
| --- | --- | --- |
| For osteogenic genes | | |
| *Runx2* | GCTTCTCCAACCCACGAATG | GAACTGATAGGACGCTGACGA |
| *OCN* | TCTTTCTCCTTTGCCTGGC | CACCGTCCTCAAATTCTCCC |
| *ALP* | AGGCAGGATTGACCACGG | TGTAGTTCTGCTCATGGA |
| *gapdh* | CCTGCACCACCAACTGCTTA | GGCCATCCACAGTCTTCTGAG |
| For adipogenic genes | | |
| *PPAR-γ* | TGGAGCCTAAGTTTGAGTTTG | ATCTTCTGGAGCACCTTGG |
| *C/EBP-α* | GAGCCGAGATAAAGCCAACAG | TCATTGTCACTGGTCAACTCCAA |

**Table S2. Sequences of synthetic TAB2-wt, TAB2-mut, CTNNB1-wt and CTNNB1-mut**

| Sequence | TAB2-wt |
| --- | --- |
| DNA sequence (5’-3’) | GATCCCACAAAGAGAAGGAACAAGGTT**TGACTATTAAAAACTCTGCTGC**CACATAGTGCCAGTAGAGGCCTACTAGGC |

The miR-214 binding sites are bolded.

| Sequence | TAB2-mut |
| --- | --- |
| DNA sequence (5’-3’) | GATCCCACAAAGAGAAGGAACAAGGTT**TGACTATTAAAAACTAGTAGGC**CACATAGTGCCAGTAGAGGCCTACTAGGC |

The miR-214 binding sites are bolded. The mutant binding sequence is shown in red.

| Sequence | CTNNB1-wt |
| --- | --- |
| DNA sequence (5’-3’) | GATCCGTTTACCAGTTGCCTTTTATCC**CAAAGTTGTTGTAACCTGCCGT**GATACGATGCTTCAAGAGGCCTACTAGGC |

The miR-214 binding sites are bolded.

| Sequence | CTNNB1-mut |
| --- | --- |
| DNA sequence (5’-3’) | GATCCGTTTACCAGTTGCCTTTTATCC**CAAAGTCACTACAACACATCGT**GATACGATGCTTCAAGAGGCCTACTAGGC |

The miR-214 binding sites are bolded. The mutant binding sequence is shown in red.

**BacECre**

**Bac214S**

**loxP**

**P_EF1-α_**

**d2EGFP**

**WPRE**

**pA**

**loxP**

**10×miR214**

**Sponges**

miR214-3p 3’-UGACGGACAGACACGGACGACA-5’

miR214 Sponges 5’-ACUGCCUGUCUGUGCCUGCUGU-3’

**loxP**

**P_EF1-α_**

**BMP2**

**WPRE**

**pA**

**loxP**

**BacLEBW**

**P_EF1-α_**

**Cre**

**pA**

**Fig. S1.** Baculovirus vectors used in this study. BacECre expressed Cre recombinase; BacLEBW expressed the potent osteogenic growth factor BMP2; and Bac214S harbored a miR-214 sponge expression cassette flanked by loxP sites. pA, polyadenylation signal; loxP, loxP recognition sequences for Cre recombinase. P_EF-1α_, EF-1α promoter derived from rat. In Bac214S, 10 tandem repeats of miR-214 sponges were cloned at the 3’ UTR of a reporter gene *d2EGFP* (destabilized enhanced green fluorescent protein). WPRE, woodchuck hepatitis virus posttranscriptional regulatory element (which stabilizes mRNA and hence enhances transgene expression). All genes were driven under the control of rat EF-1α promoter because the CMV promoter commonly used in BV constructs was weak in rat cells ( HYPERLINK \l "_ENREF_20" \o "Li, 2016 #264"
Li, et al., 2016).
